# Inflammation Spreading: Negative Spiral Linking Systemic Inflammatory Disorders and Alzheimer’s Disease

**DOI:** 10.3389/fncel.2021.638686

**Published:** 2021-02-25

**Authors:** Junjun Ni, Zhou Wu

**Affiliations:** ^1^Key Laboratory of Molecular Medicine and Biotherapy, Department of Biology, School of Life Science, Beijing Institute of Technology, Beijing, China; ^2^Department of Aging Science and Pharmacology, Faculty of Dental Science, Kyushu University, Fukuoka, Japan; ^3^OBT Research Center, Faculty of Dental Science, Kyushu University, Fukuoka, Japan

**Keywords:** systemic inflammation, macrophages, neuroinflammation, Alzheimer’s disease, cathepsin, cytokines, systemic inflammatory disorders

## Abstract

As a physiological response to injury in the internal body organs, inflammation is responsible for removing dangerous stimuli and initiating healing. However, persistent and exaggerative chronic inflammation causes undesirable negative effects in the organs. Inflammation occurring in the brain and spinal cord is known as neuroinflammation, with microglia acting as the central cellular player. There is increasing evidence suggesting that chronic neuroinflammation is the most relevant pathological feature of Alzheimer’s disease (AD), regulating other pathological features, such as the accumulation of amyloid-β (Aβ) and hyperphosphorylation of Tau. Systemic inflammatory signals caused by systemic disorders are known to strongly influence neuroinflammation as a consequence of microglial activation, inflammatory mediator production, and the recruitment of peripheral immune cells to the brain, resulting in neuronal dysfunction. However, the neuroinflammation-accelerated neuronal dysfunction in AD also influences the functions of peripheral organs. In the present review, we highlight the link between systemic inflammatory disorders and AD, with inflammation serving as the common explosion. We discuss the molecular mechanisms that govern the crosstalk between systemic inflammation and neuroinflammation. In our view, inflammation spreading indicates a negative spiral between systemic diseases and AD. Therefore, “dampening inflammation” through the inhibition of cathepsin (Cat)B or CatS may be a novel therapeutic approach for delaying the onset of and enacting early intervention for AD.

## Introduction

Alzheimer’s disease (AD), the most common type of dementia, is becoming an international public health problem with the growth of the aged population worldwide. Developing effective approaches for preventing and enacting early intervention for AD is an urgent issue, as relatively little progress in crafting new therapies to combat AD has been made. Chronic neuroinflammation is well accepted as the most relevant pathological features of AD, regulating other pathological hallmarks of AD, such as the accumulation of amyloid-β (Aβ) and hyperphosphorylation of Tau, both of which are involved in the neuronal dysfunction in AD (Carlyle et al., 2014; Chen, 2015). However, there is a great deal of evidence suggesting the important role of systemic inflammation in the pathogenesis of AD, especially in neuroinflammation. Neuroinflammation, and indeed, inflammation in general, is widely considered a major contributor to the onset as well as the pathological process of AD (Holmes, 2013). Therapies aimed at reducing systemic inflammation in individuals with mild cognitive impairment (MCI) and AD have proven beneficial by delaying the cognitive decline in these individuals, suggesting that recognition of the cross-talk between systemic inflammation and neuroinflammation has important implications for AD therapeutic strategies (Ide et al., 2016; Zhu et al., 2018).

It is well accepted that the pro-inflammatory mediators, including interleukin (IL)-1β, IL-6, and tumor necrosis factor (TNF)-α, are co-related factors involved in both systemic inflammation and neuroinflammation and affecting their sustainment and convergence. In contrast, intracellular enzymes, such as lysosomal cathepsins, mediate the production of pro-inflammatory mediators from both the periphery and brain (Voet et al., 2019).

The present review highlights the available evidence that inflammation spreading indicates a negative spiral between systemic disorders and AD. We also discuss the molecular mechanisms underlying the crosstalk between systemic inflammation and neuroinflammation. We believe that “dampening inflammation” may be a novel therapeutic approach for delaying the onset of and enacting early intervention for AD.

## Neuroinflammation in Ad

### Microglia and Neuroinflammation in AD

Making up 5%–10% of the cells in the brain, microglia are the most abundant of the resident macrophage populations in the central nervous system (CNS). Microglia are involved in surveillance and are characterized by branching with small cell bodies and long protrusions (Michell-Robinson et al., 2015). Under this homeostatic condition, microglia can secrete growth factors to support and protect the electrophysiological functions of neurons, thereby maintaining CNS homeostasis and dynamically monitoring the synaptic function through synaptic contact. Also, microglia have been reported to maintain the normal synaptic function through trimming or clearing damaged or redundant synapses (Wake et al., 2013). Following an insult or injury, microglia become activated and adopt different states, and this polarization has resulted in the common categorizations of M1-like microglia, which are suggested to have a more ‘classic’ role in the inflammatory response, and M2-like microglia, which exert an anti-inflammatory role to resolve and suppress inflammatory responses (Ni et al., 2015, 2019a; Ransohoff, 2016). The elimination and repopulation of resident microglia in aged mice significantly improved spatial memory and increased both neurogenesis and dendritic spine densities (Elmore et al., 2018). However, sustained microglial elimination impaired the parenchymal plaque development in an AD mouse model (Spangenberg et al., 2019). These observations suggest that microglia are a critical causative factor in the development and progression of aging and AD.

Neuroinflammation, an inflammatory reaction in the CNS, is usually characterized by microglial activation and collateral brain damage and is caused by a strong inflammatory reaction. The excessive activation of microglial cells resulted in the release of a large number of proinflammatory factors, which aggravated brain damages (Spangenberg et al., 2016). In addition to microglia, astrocytes, which constitute another type of glial cells in the CNS, are also involved in neuroinflammation. In a bioinformatics analysis, aging-related genes in the microglia were associated with the inflammatory response, whereas these genes in astrocytes included wildly recognized AD risk genes (Pan et al., 2020). In the progress of AD, microglia are activated and secrete neuroinflammatory factors, including IL-1β, IL-6, and TNF-α, which can kill pathogens and promote tissue repair by enhancing phagocytosis. However, the excessive release of IL-1β, IL-6, and TNF-α induces chronic neuroinflammation and aggravates brain damage (Voet et al., 2019). IL-1β, a master regulator of neuroinflammation, is a very potent signaling molecule that is expressed normally at low levels but is induced rapidly in response to local or peripheral insults (Basu et al., 2004). In AD, IL-1β, IL-6, and TNF-α are involved in causing neurodegenerative changes (Griffin et al., 1998; Paganelli et al., 2002; Uslu et al., 2012). These proinflammatory mediators are largely produced by microglia in the CNS, while exhibiting distinct activity pathways (Ni et al., 2015, 2019b).

### Neuroinflammatory Mediators and Neuronal Dysfunction

Under conditions of neuroinflammation, proinflammatory cytokines, such as IL-1β, IL-6, and TNF-α, are released by activated microglia to promote a neuroinflammatory state. The proinflammatory cytokines can either directly affect neurons or indirectly regulate the expression of many other genes and consequently affect neurons, resulting in neuronal dysfunction. In the hippocampus, high concentrations of IL-1β act on neurons to inhibit synaptic strength and long-term potentiation (LTP). The classical downstream signaling pathways are activated following IL-1β/IL-R binding. One of these pathways involves the phosphorylation and subsequent degradation of the IκB subunit of nuclear factor κB (NF-κB), leading to the nuclear translocation of NF-κB and its gene expression. Another pathway is the activation of mitogen-activated protein kinases (MAPKs), extracellular signal-regulated kinase (ERK), and p38. Among these, p38 and MAPK were reported to be activated in neurons (Srinivasan et al., 2004), while alternative signaling involving Src kinase has also been reported to be activated by IL-1β/IL-R in hippocampal neurons (Viviani et al., 2003). Primary cultured neurons exposed to recombinant IL-1β showed a significant decrease in the level of the synaptic vesicle protein synaptophysin and in the number of synaptic sites (Li et al., 2003). Also, IL-1β regulates dendritic spine morphology by upregulating the transcriptional factor methyl CpG binding protein 2 (MeCP2) in a mechanistic target of rapamycin (mTOR)-dependent manner (Tomasoni et al., 2017), leading to an excitatory/inhibitory unbalance by delaying the developmentally regulated switch of gamma-aminobutyric acid (GABA) signaling (Corradini et al., 2018). Unlike IL-1β, IL-6 can stimulate microglia and astrocytes to release a cascade of proinflammatory cytokines and acute-phase proteins, such as C-reactive protein (CRP; Querfurth and Laferla, 2010). In the hippocampus, the distribution of IL-6 receptor (IL-6R) is modest in glia but prominent in perikaryon and outlining the apical dendrites in neurons. IL-6R subunits in the rat cerebral cortex showed that both IL-6R and its subunit are localized in pre-and postsynaptic membranes (D’arcangelo et al., 2000). Several downstream signaling partners are associated with IL-6R activation, including Janus kinase (JAK), signal transducer and activator of transcription 3 (STAT3), MAPK, and phosphoinositide 3-kinases (PI3K), which are located with postsynaptic density at hippocampal synapses (Nicolas et al., 2012; Murase and Mckay, 2014). Therefore, IL-6/IL-6R and associated signal transduction molecules are appropriately positioned to influence the synaptic function. TNF-α interacts with two cognate receptors, TNF receptor (TNF-R) I and TNF-RII. These receptors are expressed on various cell types, including neurons, throughout the CNS. The binding of homotrimeric TNF-α to either receptor can activate three major signaling cascades: the Fas/caspase-8, NFκB, and c-Jun N-terminal kinase (JNK) pathways (Park and Bowers, 2010). TNF-RI is a member of the death receptor family, the activation of which may result in the death of neurons.

### Neuroinflammatory Mediators and AD Pathologies

In AD models, the neuroinflammatory cytokines have been found to modulate the expression of amyloid precursor protein (APP), which leads to increased Aβ production. A previous study reported that exposure to IL-1β increased the mRNA expression of APP in neuronal cells (Forloni et al., 1992). Similarly, exposure to TNF-α upregulated APP in both neurons and astrocytes. Neuroinflammatory cytokines have also been shown to increase APP metabolic enzymes, including the β- and γ-secretase enzymatic activity (Yamamoto et al., 2007). In addition to evidence concerning the effects of neuroinflammation on the APP and its processing in AD, neuroinflammation has also been reported to affect Tau tangle formation. A recent study suggested that hippocampal synaptic pathology and microgliosis might be the earliest manifestations of neurodegeneration related to tauopathies, and indeed, immunosuppression in the AD model mice diminished the tau pathology and increased the animal’s lifespan (Yoshiyama et al., 2007). Activated microglia may promote neurodegeneration; however, they also play neuroprotective roles. For example, local chronic upregulation of microgliosis in AD mice ameliorated plaque pathology, and brain microgliosis induced by the peripheral administration of colony-stimulating factor led to the attenuation of Aβ plaque and improved learning and memory in AD mice (Shaftel et al., 2007; Boissonneault et al., 2009). The dual effects of microglia-induced neuroinflammation may be attributed to a diverse set of “activation” phenotypes—what one may call the “two faces” of disease-associated microglia. The first face is an immunosuppressive phenotype with an anti-inflammatory function and which is involved in the phagocytosis of Aβ. This type of microglia is seen as a defensive or neuroprotective factor linked to amyloid clearance (Cherry et al., 2015; Onuska, 2020). The other face of microglia appears in more advanced stages of AD and has been shown to cause extensive inflammation and neurodegeneration. Nevertheless, the sustained overexpression of IL-1β exacerbated tau pathology despite a reduced amyloid burden in AD mice (Ghosh et al., 2013). Although the roles of microglia in the initiation and progression of AD are heavily debated, other reports describe the protective contribution of microglia to AD. Thus, the involvement of microglia in AD pathologies has generated strong interest.

The presence of neuroinflammation-accelerated neuronal dysfunction in AD may influence the functions of the peripheral organs. It is considered that neuronal dysfunction in AD negatively impacts the peripheral organs through peripheral Aβ accumulation; high levels of Aβ has been found in the peripheral circulation of AD patients; this is attributed to a reduction of peripheral Aβ clearances as well as peripheral Aβ production (Nie et al., 2019; Gu et al., 2020). Bone is influenced by high peripheral levels of Aβ. It is reported that Aβ induces the differentiation and activation of osteoclasts, resulting in the promotion of bone destruction (Cui et al., 2011; Li et al., 2016). Indeed, neuronal dysfunction with the brain structure changes in AD has been suggested to reduce the bone mass (Loskutova et al., 2010, 2019). This is supported by the high incidence of fracture in AD patients with a low bone mineral density (Friedman et al., 2010; Wang et al., 2014). IL6 and IL17 are considered to be the factors linking bone destruction and neuronal dysfunction in AD (Gu et al., 2020). The gut is influenced by peripheral Aβ. It is suggested that peripheral Aβ promotes the intestinal inflammatory process, resulting in a reduction in the barrier function of the gut (Wang et al., 2017). Recent research suggests that other peripheral organs, such as the liver and gums are also influenced by peripheral Aβ. Aβ increases macrophages to produce IL-1β to enhance systemic inflammation, resulting in a reduction in the phagocytosis ability of macrophages, which delays recovery from the pathology (Nie et al., 2019). The impact of other pathologies of AD in the peripheral organs needs to be explored in further studies.

## Systemic Inflammatory Disorders and Ad

Chronic systemic inflammatory conditions may be associated with increased AD risk and accelerated AD progression (Perry and Holmes, 2014), and a positive link between systemic inflammation and AD has been considered through the deregulation of the inflammatory cascade.

### Bone-Related Inflammation and the Risk of AD

Rheumatoid arthritis (RA), the most common inflammatory bone disease, has a prevalence of approximately 1% among adults, and RA patients have higher levels of systemic inflammation than those without RA (Mason et al., 2018). A relationship between RA and AD has been reported since the early 1990s (Mcgeer et al., 1990). A meta-analysis including 17 epidemiological studies demonstrated that Non-Steroidal Anti-Inflammatory Drugs (NSAIDs) protect against AD onset (Mcgeer et al., 1996), and a prospective study of 7,000 healthy subjects showed that the long-term use of non-steroidal anti-inflammatory drugs (NSAIDs) protected against AD development (In T’ Veld et al., 2001). A systematic review of multiple prospective and non-prospective studies further showed that NSAID exposure was associated with a decreased risk of AD (Szekely et al., 2004). More recently, research has shown that usage of classical disease-modifying antirheumatic drugs, especially methotrexate, reduced the AD risk in RA patients (Judge et al., 2017). Indeed, the incidence of AD in RA patients is higher than in the general population (Lin et al., 2018), which was consistent with the finding that AD was more prevalent among RA patients than among those without RA in a nested case-control study analyzing more than 8.5 million commercially insured adults (Chou et al., 2016). A 21-year follow-up of the association between RA or arthritis and dementia/AD in several case-control and hospital- and register-based studies further showed that the presence of joint disorders, especially RA, in midlife appears to be associated with cognitive decline in later life (Wallin et al., 2012).

### Oral Inflammation and the Risk of AD

Periodontitis, the most common chronic oral inflammatory disorder in adults with alveolar bone loss, has been recognized as a risk factor for AD (Kamer et al., 2008) since morbidity of periodontitis is positively correlated with both the onset and pathological progression of AD clinically (Kamer et al., 2015; Ide et al., 2016). *Porphyromonas gingivalis* (*P. gingivalis*), a keystone pathogen for periodontitis, is recognized as the major linking factor between periodontitis and AD since *P. gingivalis* DNA as well as its virulence factors, including lipopolysaccharide (LPS) and gingipain, have been detected in the cortex and cerebrospinal fluid (CSF) of AD patients (Poole et al., 2013; Dominy et al., 2019), and antibodies to *P. gingivalis* were shown to be elevated in the serum of AD patients (Kamer et al., 2015). Recent preclinical studies have suggested that periodontitis may contribute to the onset of AD, as exposure to *P. gingivalis* and its LPS induced hallmarks of AD-like pathology, including Aβ accumulation, neuroinflammation, and memory decline, in middle-aged mice (Wu et al., 2017; Nie et al., 2019; Gu et al., 2020; Zeng et al., 2020). Therefore, bone-related inflammation may increase the risk of AD (Wu and Nakanishi, 2015).

### Gut Inflammation and the Risk of AD

Gut inflammation and dysbiosis are directly associated with gut barrier dysfunction and increased intestinal permeability, and the interruption of the barrier causes the translocation of bacteria and the introduction of harmful substances into the bloodstream, resulting in systemic inflammation (Bischoff et al., 2014; König et al., 2016). An abundance of mucin-degrading bacteria *Akkermansia muciniphila* is known to improve the gut barrier function and reduce systemic inflammation (Alkasir et al., 2017), and probiotic strains, such as *Lactobacillus Plantarum*, *Escherichia coli Nissle*, and *Bifidobacterium infantis*, enhance the intestinal barrier, increasing the expression of proteins that form tight junctions (Bischoff et al., 2014). In contrast, *E. coli* strains, *Salmonella*, and *Escherichia*/*Shigella* *Clostridium* mediate intestinal permeability by reducing the formation of tight junctions (König et al., 2016). The influence of gut microbiota on AD has been investigated. A recently conducted study revealed that the increased abundance of proinflammatory *Escherichia* *Shigella* and decreased abundance of anti-inflammatory *Eubacterium rectale* might be associated with systemic inflammation in AD patients (Cattaneo et al., 2017). Another study showed that the fecal microbiota profile in AD patients was characterized by reduced microbial diversity, a decreased abundance of *Firmicutes* and *Bifidobacterium*, and an increased abundance of *Bacteroidetes* (Vogt et al., 2017). The relative bacterial abundance correlated with the increase in CSF markers of AD pathology (Vogt et al., 2017). In addition to alterations in the gut microbiota composition, the increased number of bacteria in the small intestine also influences the permeability, which has been seen in AD patients (Kowalski and Mulak, 2019). Gut microbiota may play a regulatory role in the pathogenesis of AD (Szablewski, 2018), with the relative bacterial abundance correlating with increases in CSF markers of AD pathology (Vogt et al., 2017). Increased intestinal permeability can lead to an approximately three-fold increase in levels of serum LPS in AD patients compared to healthy controls (Zhang et al., 2009). Individuals with irritable bowel syndrome, a condition characterized by microbial dysbiosis, are at a 1.8-fold greater risk for developing AD than the general population (Chen et al., 2016), while ulcerative colitis patients have a 2.4-fold higher rate of mortality due to AD (Caini et al., 2016). DW2009, a *Lactobacillus Plantarum* C29-fermented soybean, was found to improve cognitive performance in individuals with mild cognitive impairment (MCI) in a double-blind, randomized clinical trial. Despite the possible large variance in gut microbiota composition between individuals, the consumption of DW2009 significantly increased the number of lactobacilli in the gut bacterial composition through stimulation of the proliferation of the gut lactobacilli population (Hwang et al., 2019). Therefore, the microbial dysbiosis that causes increased barrier permeability and systemic inflammation may act synergistically to accelerate AD pathology.

### Infection and the Pathologies in AD

**Figure 1:**
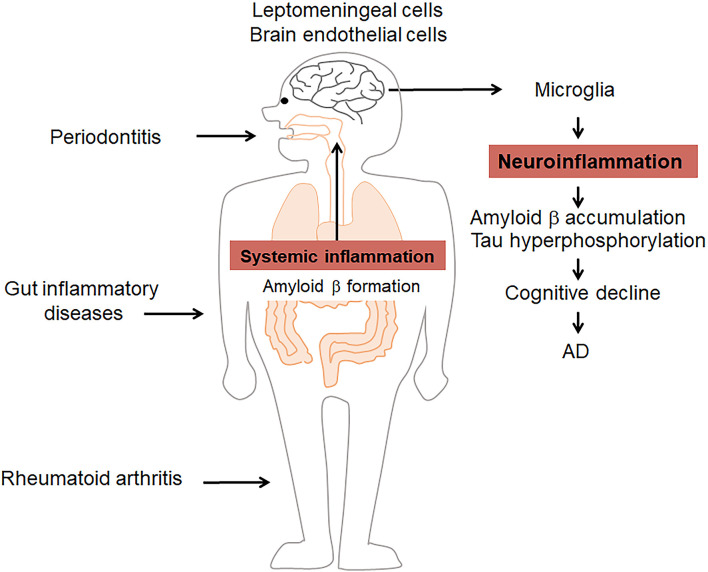
Systemic inflammatory disorders influence Alzheimer’s disease (AD) by amplifying inflammation. Periodontitis, rheumatoid arthritis (RA), and gut inflammatory diseases induce/amplify systemic inflammation as well as amyloid β (Aβ) formation to induce/prolong pathological changes in the brain, including microglia-related neuroinflammation, Aβ formation, and tau hyperphosphorylation. These events contribute to the cognitive decline in AD.

It has been demonstrated that brain infection with herpes simplex virus type 1 (HSV1), *Chlamydia pneumonia*, spirochetes as well as fungal in AD patients (Hammond et al., 2010; Itzhaki, 2014; Miklossy, 2015; Pisa et al., 2015), suggesting that microbial infection in the etiology of AD, which is advocated as pathogen theory of AD (Itzhaki et al., 2016). More recently, research has shown that *P. gingivalis* infection of the brain is involved in the pathology of AD (Liu et al., 2017; Dominy et al., 2019). The routes of microbial infection of the brain have been discussed. The olfactory nerve, which leads to the lateral entorhinal cortex, is a route of entry of HSV1 as well as *Chlamydia pneumonia* into the brain (Little et al., 2005; Mori et al., 2005), which is supported by olfactory dysfunction is an early symptom of AD. Indeed, the olfactory bulb has been known as the initial site from which characteristic AD pathology subsequently spreads through the brain (Ball et al., 2013). The brainstem is known to harbor latent HSV, and brainstem virus reactivation would affect AD (Braak and Del Tredici, 2015). Brain microbial infection is involved in the pathology of AD through the induction of neuroinflammation and brain Aβ accumulation. It has been reported that brain infection by *P. gingivalis* provokes gingipain-dependent neuroinflammation by increasing microglial migration to produce IL-6 and TNF-α (Liu et al., 2017), inducing neurotoxic effects. This is demonstrated by the finding that gingipain inhibition reduced *P. gingivalis* brain infection and neuroinflammation, resulting in the blocking of Aβ_1–42_ production (Dominy et al., 2019). Moreover, *P. gingivalis* LPS has been found to induce the neuroinflammation-dependent cellular accumulation of Aβ_1–42_ in neurons, and thus to contribute to memory decline in mice (Wu et al., 2017). These observations suggest that the prevention of infection could be beneficial for delaying neurodegeneration in AD. However, Aβ is also known as an antimicrobial peptide with potent activity against multiple bacteria and viruses, even in the brain (Bourgade et al., 2015; Kumar et al., 2016). Taken together, these findings suggest that systemic inflammatory disorders influence AD by amplifying inflammatory cascades (Figure 1).

## Transfer Routes of Systemic Inflammatory Signals to Neuroinflammation

Systemic inflammation can initiate or exacerbate brain pathology, even in the absence of the overt invasion of bacteria into the brain. Therefore, cellular transfer routes of systemic inflammatory signals into the brain have been intensively investigated. According to previous studies, these routes are as follows: (1) a direct pathway through the circumventricular organs, which lack a blood-brain barrier (BBB); (2) activation of the brain endothelial cells; (3) activation of a transporter across the BBB; and (4) interaction with the vagus nerve (Perry et al., 2003). In addition to the above classical routes, leptomeningeal cells, which cover the cortex of the brain, are considered a new route between systemic inflammation and neuroinflammation, as leptomeningeal cells produce proinflammatory mediators to induce neuroinflammation (Wu et al., 2005, 2007; Liu et al., 2013).

### The BBB

The BBB is composed of specialized endothelial cells, glial cells, pericytes, and a basement membrane that prevents immune cell migration and soluble molecule diffusion from the circulation into the CNS, such as the brain (Perry et al., 2003; Perry, 2004). Brain endothelial cells are responsible for transferring systemic signals into the brain, as these cells express functionally significant amounts of receptors, including Toll-like receptor (TLR)4, and are capable of producing inflammatory mediators (Danese et al., 2007). Repeated systemic LPS challenges, a simple model of systemic inflammation, reportedly induce the rapid upregulation of the expression of cell adhesion molecules, including vascular cell adhesion protein (VCAM)-1 and intercellular adhesion molecule (ICAM)-1, as well as the prolonged upregulation of major histocompatibility complex (MHC) I and MHCII expression on cerebral capillaries. LPS induces long-lasting changes to cerebral vasculature for BBB permeability, resulting in the induction of neuroinflammation (Puntener et al., 2012).

### Brain Endothelial Cells and Their Transporter

Systemic inflammation induced by bacteria and LPS alters the Aβ transport in brain endothelial cells, resulting in brain Aβ accumulation (Jaeger et al., 2009; Erickson et al., 2012; Zeng et al., 2020). Low-density lipoprotein receptor-related protein-1 (LRP-1) and p-glycoprotein (Pgp) are the efflux transporter of Aβ, while receptor for advanced glycation endproducts (RAGE) is the influx transporter for Aβ across the BBB (Deane et al., 2003, 2004). Researchers have found that the systemic administration of LPS inhibits Aβ efflux transport out of the brain by downregulating the expression of both LRP-1 and Pgp in the brain endothelial cells (Jaeger et al., 2009; Erickson et al., 2012). In contrast, we recently found that the systemic administration of *P. gingivalis* promotes Aβ influx into the brain by upregulating the expression of RAGE in brain endothelial cells (Zeng et al., 2020).

Moreover, ligation of RAGE triggers a series of cellular signaling events, including the activation of NF-κB, leading to the production of proinflammatory cytokines, and causing inflammation. RAGE could also activate MAPK signaling cascades, which thereby release and activate NF-κB downstream (Wang et al., 2020). The fact that RAGE-knockout mice appear to be healthy and developmentally normal suggests RAGE inhibition to be a safe therapeutic approach. FPS-ZM1, a high-affinity RAGE-specific inhibitor, has been reported to effectively control neuroinflammation and the progression of Aβ-mediated neurodegeneration. These observations further support the notion that the activation of transport into brain endothelial cells transfers systemic inflammatory signals for inducing or promoting lesions in the brain. A recent study showed that systemic inflammation induced neuroinflammation, in turn contributing to BBB dysfunction (Haruwaka et al., 2019).

### Vagus Nerve

The vagus nerve (VN) transfers systemic inflammatory signals into the brain, which is involved in modulating neuroinflammation. Specifically, 80% of the VN is composed of sensory afferent fibers (Berthoud and Neuhuber, 2000) and primary sensory afferents have been proposed to serve as an anatomical substrate conveying systemic inflammatory messages, triggered by locally inflammatory mediators, to the brain (Watkins et al., 1995). Activation of glutamate afferents in the brainstem medulla oblongata has been considered as the first step of the involvement of the VN in the communication pathway from the systemic LPS and IL-1β to the brain (Mascarucci et al., 1998) because A2 neurons are located within the nucleus tractus solitaries (located in the brainstem and medulla oblongata), which receive direct synaptic contacts from the vagal afferents (Herrera-Marschitz et al., 1996). As the longest nerve in the body, the VN is distributed in the oral cavity, neck, internal chest, and the internal abnormal organs. It transfers systemic inflammatory signals from the organs to which it is distributed into the brain, including the prefrontal cortex (Buijs and Van Eden, 2000). Because the TLR4 are expressed on VN afferent fibers (Goehler et al., 1999), VN afferent fibers can sense bacterial products, such as LPS, to activate the brain (Lal et al., 2001). On the other hand, anti-inflammatory effects have been shown after VN activation. A recent report showed that VN stimulation prevented systemically LPS-induced hippocampal microglial activation-related neuroinflammation by reducing ionized calcium-binding adapter molecule 1 (Iba-1) immunoreactivity, resulting in the amelioration of cognitive dysfunction in mice (Huffman et al., 2019). Also, VN stimulation attenuated the upregulation of IL-6 and TNF-α in the brainstem of rat pups caused by the systemic administration of LPS (Johnson et al., 2016). The anti-inflammatory effects of the VN may be related to acetylcholine, the principal vagal neurotransmitter, because acetylcholine can attenuate the release of IL-1β, TNF-α, and IL-6 in LPS-stimulated cells (Borovikova et al., 2000). Therefore, VN afferents transfer systemic inflammatory messages to the brain and may also be involved in the reduction in neuroinflammation.

### Leptomeningeal Cells

Leptomeningeal cells transduce systemic inflammatory signals into the brain by producing IL-1β, TNF-α, and IL-6 (Wu et al., 2005, 2007, 2008), as leptomeningeal cells express TLR2 and TLR4 that could bind to bacteria and their components (Liu et al., 2013). Leptomeningeal cell-produced IL-1β is involved in the reduction in the expression of tight junction proteins, including occludin, resulting in the amplification of systemic inflammatory signals to enhance neuroinflammation (Wu et al., 2008). Using an *in vitro* cellular culture system, we found that the production of IL-1β and TNF-α in leptomeningeal cells was induced by treatment with the conditioned medium from *P. gingivalis* LPS-stimulated macrophages; the levels of these proteins were significantly higher than those induced by treatment with *P. gingivalis* LPS alone. Also, the production of IL-1β and TNF-α in microglia was upregulated by treatment with the conditioned medium from *P. gingivalis* LPS-treated leptomeningeal cells, resulting in levels were significantly higher than those induced by treatment with *P. gingivalis* LPS alone (Liu et al., 2013). These previous findings indicate that leptomeningeal cells transfer systemic inflammatory signals from macrophages to induce microglia-related neuroinflammation in response to systemic inflammatory signals.

Taken together, the above evidence suggests that chronic inflammation associated with systemic inflammatory disorders is involved in promoting the initiation and pathological processes of AD.

## Co-Related Pro-Inflammatory Molecules Between Systemic Inflammation and Neuroinflammation

Since inflammation is a common feature of both AD and systemic inflammatory disorders, the existence of co-related pro-inflammatory molecules could explain the link between systemic inflammatory disorders and AD. Understanding the inflammatory cascade pathways would thus help identify effective approaches for regulating AD. As mentioned above, inflammation is a common feature of both AD and systemic inflammatory disorders. Pro-inflammatory cytokines, such as IL-1β, IL-6, and TNF-α, are representative co-related molecules involved in the induction and regulation of the inflammatory cascade in systemic inflammatory disorders, including RA, periodontitis, and gut inflammatory diseases.

### IL-1β

IL-1β is a critical inducer of pathogenesis and tissue damage. It is considered to induce inflammatory bone disorders, including RA and periodontitis (Zwerina et al., 2007; Kim et al., 2009) since its presence reduces the activation of osteoblasts (Hengartner et al., 2013) but increases the activation of osteoclasts (Kulkarni et al., 2012). Clinically, IL-1β is used as a biomarker to assess the therapeutic outcomes of patients with chronic periodontitis (Buduneli and Kinane, 2011). Elevated IL-1β levels are associated with an increase in inflammatory bowel disease (IBD) severity (Coccia et al., 2012). The involvement of IL-1β in gut inflammation has been evidenced by the fact that deletion of IL-1β from monocytes consequently attenuated dextran sulfate sodium (DSS)-induced colitis (Seo et al., 2015), and direct inhibition of IL-1β signaling reduced intestinal inflammation in DSS-induced colitis with the chronic granulomatous disease (De Luca et al., 2014). IL-1β is thus considered to act in conjunction with IL-6 and TNF-α to induce inflammation in IBD (Mao et al., 2018).

### IL-6

IL-6, a pro-inflammatory cytokine with pleiotropic biological activities, plays a key role in RA (Kim et al., 2015) and periodontitis (Nibali et al., 2012). IL-6 contributes to the induction and maintenance of the inflammatory process by promoting T-helper 17 (Th17) cell differentiation, which is involved in bone distribution (Navarro-Millán et al., 2012). Also, IL-6 is important due to its involvement in IBD, and anti-IL-6 agents have been used clinically for RA and IBD treatment (Kim et al., 2015). A cohort study showed that the serum IL-6 levels significantly increased from about 5 years before the onset of AD (Tilvis et al., 2004), and elevated serum IL-6 levels in middle age increased the risk of developing AD in the next 10 years by about 2-fold (Singh-Manoux et al., 2014). Furthermore, IL-6 contributes to the induction and maintenance of the inflammatory process by promoting Th17 differentiation (Navarro-Millán et al., 2012; Dekita et al., 2017; Gu et al., 2020).

### TNF-α

As a key molecule involved in the induction and maintenance of inflammation, TNF-α induces local inflammation, resulting in bone destruction in RA and periodontitis by enhancing the receptor activator of nuclear factor kappa-B ligand (RANKL) expression (Marahleh et al., 2019). The application of therapies targeting TNF-α has considerably improved the treatment of RA (Chou et al., 2016). A clinical study showed that the serum levels of TNF-α were associated with cognitive decline in AD patients. An increase in the serum levels of TNF-α resulted in a two-fold increase in the rate of cognitive decline over 6 months, and high baseline levels of TNF-α were associated with a four-fold increase in the rate of cognitive decline (Holmes et al., 2009). Our recent clinical research showed that the intake of propolis, a resinous mixture produced by honey bees, for 2 years prevented the cognitive decline in elderly individuals by decreasing the serum levels of IL-1β, IL-6, and TNF-α. This suggests that systemic inflammation may be a potential target for preventing AD and developing new therapies (Zhu et al., 2018).

Preclinical studies have shown that systemic inflammation induces age-dependent differential microglia-related neuroinflammation. Using a stable chronic inflammatory rat model of adjuvant arthritis (AA), we found that microglia in the proximity of the leptomeninges produce anti-inflammatory cytokines, such as IL-10 and transforming growth factor-β1 (TGF-β1), in young adult AA rats (Wu et al., 2005, 2007, 2008). In contrast, microglia, close to the leptomeninges in middle-aged AA rats produce pro-inflammatory mediators, including IL-1β and to a lesser degree IL-10 and TGF-β1, indicating that microglia are primed even in middle age by “microglia aging” (Nakanishi and Wu, 2009). Evidence of microglial aging was first identified in the brains of aged individuals based on morphological and immunohistochemical analyses, the aging microglia have been found to have an altered surveillance phenotype with less dendritic branching and reduced process motility, and to exhibit lower migration rates and more sustained pro-inflammatory responses to injury or infection (Damani et al., 2011). The alterations in dynamic microglial behavior reveal an aspect of the microglial aging phenotype wherein senescent microglia may drive features of disease progression in the aging nervous system. A comparison of single-nucleus transcriptomics between humans and mice revealed a remarkable difference. For example, variants of the microglial receptor TREM2 increase the AD risk and activation of “disease-associated microglia” (DAM) is dependent on TREM2 in AD mice. In human AD, the microglia acquire a quite distinct signature characterized by the increased expression of “homeostatic” genes along with genes that are absent in the DAM signature (Zhou et al., 2020). Using single-cell RNA sequencing of live microglia purified from the human cerebral cortex, researchers have found at least one subset of cortical microglia that may be related to AD (Olah et al., 2020). Thus, the subset of cortical microglia related to AD should be prioritized in further validation efforts.

### Cellular Enzymes for Regulating Co-related Pro-inflammatory Molecules

**Figure 2:**
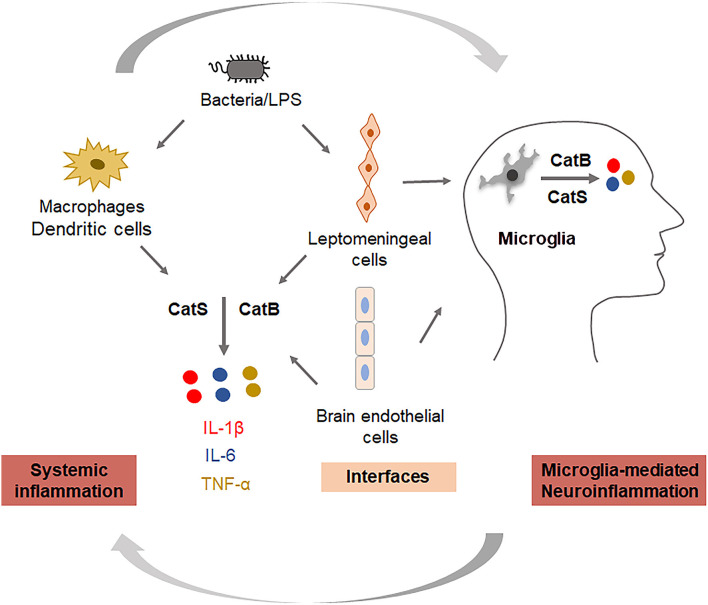
Key roles of cathepsins in regulating systemic inflammation and neuroinflammation. Cathepsin B is involved in the production of IL-1β and TNF-α, and cathepsin S is involved in the production of IL-6 in systemic cells (monocytes/macrophages), interfacing cells (leptomeningeal cells, brain endothelial cells), and neural cells (microglia).

Cathepsin (Cat) B (EC 3.4.22.1) is a lysosomal cysteine protease that is expressed constitutively and which is linked to general protein turnover in lysosomes. The expression and activity of CatB have been reported to be associated with several pathologies, including Alzheimer’s disease. In addition to its role in the cleavage of amyloid precursor protein at the wild-type β-secretase site (Hook et al., 2008), its regulation of neuroinflammation has also received attention. CatB has been primarily reported to leak from lysosomes and subsequently trigger the activation of the NLR family pyrin domain containing 3 (NLRP3) inflammasomes in microglia after phagocytosis of Aβ (Halle et al., 2008). Besides, it has also been reported to be involved in the activation and processing of pro-caspase-1 and pro-IL-1β in an NLRP3-independent manner (Terada et al., 2010). Since inflammasomes play either causative or contributing roles, and also exaggerate the pathology in responses to host-derived factors, the inhibition of CatB may dampen neuroinflammation through the inactivation of NLRP3 inflammasomes. In studies using aged or middle-aged mice, CatB was found to activate NFκB by degrading inhibitory κBα, an endogenous inhibitor of NFκB, *via* an autophagic-lysosome pathway and mitochondrial oxidative stress (Wu et al., 2017; Ni et al., 2019a), and the persistent activation of NFκB induced chronic neuroinflammation, which can damage neurons through highly toxic pro-inflammatory mediators and trigger the breakdown of the BBB, resulting in the migration of peripheral immune cells into the CNS. Also, the production of Aβ_1–42_ and Aβ_3–42_ was inhibited by pretreatment with a CatB inhibitor in macrophages after *P. gingivalis* infection. This suggests that increased levels of Aβ in the circulation may be further transported to the CNS, as *P. gingivalis* infection induced the upregulation of receptor for the advanced glycation end product in cerebral endothelial cells, a process that is CatB-dependent (Nie et al., 2019; Zeng et al., 2020). CatB is also involved in the trafficking of TNF-α-containing vesicles to the plasma membrane in macrophages (Ha et al., 2008). Taken together, CatB plays critical roles in the regulation of inflammation and the inflammatory crosstalk between the periphery and the CNS *via* the BBB; thus, CatB appears to be a target through which the ‘dampening inflammation’ may occur (Figure 2).

CatS (EC 3.4.22.27) is also a lysosomal cysteine protease with routine physiological digestion functions in antigen-presenting cells (APC). It was reported that cortical microglia contain an intrinsic molecular clock and exhibit the circadian expression of CatS. The genetic ablation of CatS in mice induced hyper locomotor activity due to failure to reduce the synaptic strength during sleep (Hayashi et al., 2013). Disturbed microglial circadian rhythms induced chronic inflammation in the brain (Ni et al., 2019b), and the increased expression of CatS was also found in the brains of AD mice, suggesting that CatS may indirectly induce neuroinflammation *via* disturbance of the microglial circadian clock. Additionally, CatS-mediated cleavage of protease-activated receptor-2 (PAR2) results in its activation and can cause the occurrence of inflammation in mice (Zhao et al., 2014). We previously found that CatS played a critical role in driving splenic DC-dependent Th17 polarization through the upregulation of IL-6 by activating PAR2 after exposure to components of periodontal bacteria (Dekita et al., 2017). Therefore, the inhibition and direct targeting of CatS could be useful for the treatment of inflammation-associated pathological processes in AD (Figure 2).

## Clinical Trials of Anti-Inflammatory Treatment

On November 2, 2019, Green Valley announced that sodium oligomannate (GV971), a marine-derived oligosaccharide, had received conditional marketing approval in China to improve the cognitive function in patients with mild to moderate AD. GV971 was reported to restore the gut microbial profile to normal and to lessen brain immune cell infiltration and inflammation in AD mice (Wang et al., 2019), and was demonstrated to improve the cognitive function in patients with mild-to-moderate Alzheimer’s disease as early as week 4 in a phase III trial. Therefore, targeting the gut microbiota proved to be an effective and feasible therapy for AD. Moreover, small molecule gingipain inhibitor ameliorated infection, reduced Aβ42 and neuroinflammation, and protected neurons from gingipain toxicity (Dominy et al., 2019). Cortexyme Inc. has completed phase 1 clinical trials of COR388 (a gingipain inhibitor) and will run a phase 2/3 study to determine whether it can improve cognition in patients with mild to moderate AD. In addition to interventions targeting the microbiota or periodontal pathogens, some small molecules directly targeting inflammation are being investigated in phase 2/3 trials, including ALZT-OP1 from AZTherapies Inc., Azeliragon from Pfizer, Epigallocatechin Gallate from Taiyo International, Etanercept from Amgen Inc., and Thalidomide from Celgene Corporation.

On the other hand, recently, in a 2-year double-blind randomized placebo-controlled trial that enrolled 195 cognitively intact elderly participants with a family history of AD, low-dose naproxen did not significantly reduce the progression of the Alzheimer Progression Score (APS), which is a composite indicator of pre-symptomatic AD (Meyer et al., 2019). This work suggested the reconsideration of inflammatory disease as a possible explanation for the reduced incidence of AD among NSAID users in observational studies. Even naproxen had no benefit on any of the APS components, and to date anti-amyloid treatment strategies for AD have largely shown unimpressive results. Thus, interest in alternative therapeutic targets, including anti-inflammatory strategies, is likely to continue to increase in the coming years.

## Conclusion

In conclusion, evidence shows that inflammation is the source of linkage between systemic diseases and AD. AD is considered a multifactorial disease affected by the negative spiral of spreading inflammation. Therefore, “dampening inflammation” may be useful as a novel therapeutic approach for delaying the onset of and enacting early intervention for AD. Inhibition of CatB and CatS, in particular, maybe an effective target for dampening inflammation.

## Author Contributions

JN and ZW conceived and drafted the manuscript. All authors contributed to the article and approved the submitted version.

## Conflict of Interest

The authors declare that the research was conducted in the absence of any commercial or financial relationships that could be construed as a potential conflict of interest.
